# Quality of obstetric care in the sparsely populated sub-arctic area of Norway 2009–2011

**DOI:** 10.1186/1471-2393-13-175

**Published:** 2013-09-14

**Authors:** Jan Norum, Anca Heyd, Bente Hjelseth, Tove Svee, Fred A Mürer, Randi Erlandsen, Barthold Vonen

**Affiliations:** 1 Northern Norway Regional Health Authority trust, Bodø, N-8038, Norway; 2 Institute of Clinical Medicine, Faculty of Health Sciences, University of Tromsø, Tromsø, N-9037, Norway; 3 Department of Oncology, University Hospital of North Norway (UNN), Tromsø, N-9038, Norway; 4 Helgeland hospital trust, Mo i Rana, N-8600, Norway; 5 Nordland hospital trust, Bodø, N-8092, Norway

**Keywords:** Quality, Obstetrics, Norway, Registry, Decentralized care, Birth weight, Infection

## Abstract

**Background:**

It is challenging to obtain high quality obstetric care in a sparsely populated area. In the subarctic region of Norway, significant distances, weather conditions and seasonable darkness have called for a decentralized care model. We aimed to explore the quality of this care.

**Methods:**

A retrospective study employing data (2009–11) from the Medical Birth Registry of Norway was initiated. Northern Norwegian and Norwegian figures were compared. Midwife administered maternity units, departments at local and regional specialist hospitals were compared. National registry data on post-caesarean wound infection (2009–2010) was added. Quality of care was measured as rate of multiple pregnancies, caesarean section, post-caesarean wound infection, Apgar score <7, birth weight <2.5 kilos, perineal rupture, stillbirth, eclampsia, pregnancy induced diabetes and vacuum or forceps assisted delivery. There were 15,586 births in 15 delivery units.

**Results:**

Multiple pregnancies were less common in northern Norway (1.3 vs. 1.7%) (*P* = 0.02). Less use of vacuum (6.6% vs. 8.3%) (*P* = 0.01) and forceps (0.9% vs 1.7%) (*P* < 0.01) assisted delivery was observed. There was no difference with regard to pregnancy induced diabetes, caesarean section, stillbirth, Apgar score < 7 and eclampsia. A significant difference in birth weight < 2.5 kilos (4.7% vs. 5.0%) (*P* < 0.04) and perineal rupture grade 3 and 4 (1.5% vs. 2.3%) (*P* < 0.02) were revealed. The post-caesarean wound infection rate was higher (10.5% vs. 7.4%) (*P* < 0.01).

**Conclusion:**

Northern Norway had an obstetric care of good quality. Birth weight, multiple pregnancies and post-caesarean wound infection rates should be further elucidated.

## Background

Northern Norway covers almost half of Norway’s land mass and is about two-thirds of the size of the UK. The population is only 470.000 inhabitants, one fourth of whom live in the two main cities (Bodø and Tromsø). About 2,500 people live in the Norwegian arctic, mainly on the Svalbard islands. Significant distances have been a constant challenge to the northern Norwegian specialised health care in terms of quality of care, costs and logistics. The area has a subarctic and arctic climate causing great challenges, especially during winter time. Cold and rough weather conditions, long distances, seasonable darkness and snow have to be handled.

Maternity care and delivery may be organized differently in sparsely populated areas. In northern Norway, midwife administered maternity units (MAMUs) have been an important part of health care [1,2]. To secure a high quality of care, Norwegian women have been selected to institution/unit of delivery according to risk factors [1,2]. Generally, three levels of care are available. Those at low risk may deliver at MAMUs [2]. Women with intermediate risk are referred to second-level perinatal care units at local hospitals and those with high risk to a regional specialist hospital for delivery. Whereas the MAMUs generally are staffed with midwives and have a general practitioner on duty available in the community, they have no gynaecologist/obstetrician. The second level (department of obstetrics and gynaecology) has such a specialist on duty 24 hours a day and emergency caesarean section may be performed at any time. The regional specialist hospitals are superior to the departments at local hospitals in terms of access to paediatricians on duty and a paediatric intensive care unit.

The quality of maternal health care services in Norway has been scrutinised recently. The Ministry of Health and Care Services launched a plan for improved maternity and delivery care entitled ‘A Happy Occasion’ and incorporated it in the ’Coordination Reform’ [3,4]. The main aim of this document is to improve continuous maternity, delivery and postpartum care. The changes to selection criteria and procedures for transportation will result in more centralised care. In this context, in March 2012, we initiated the national registry-based study to analyse the de-centralised obstetric care currently (2009–2011) provided in Northern Norway.

## Methods

**Figure 1:**
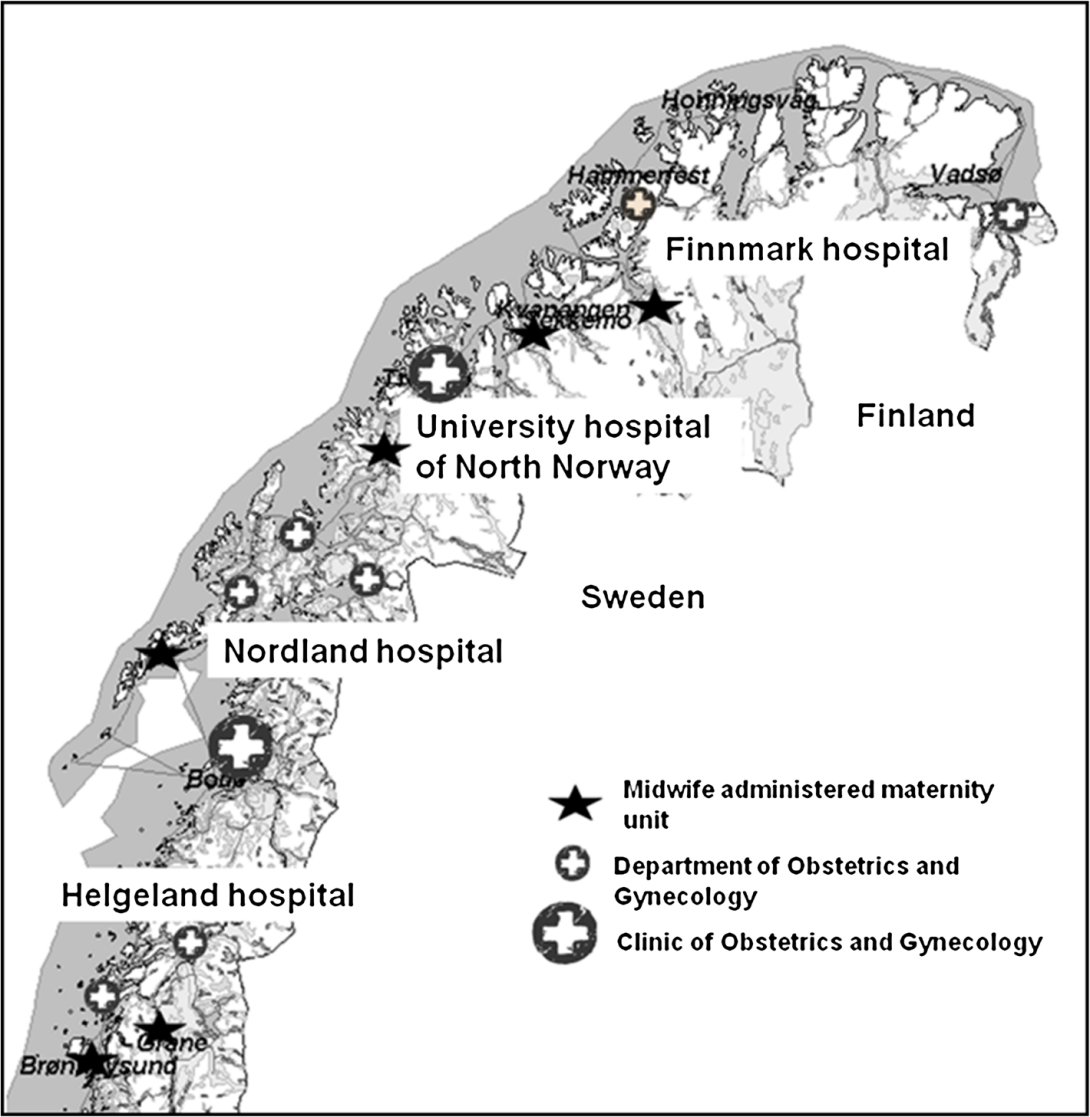
The figure shows the midwife administered maternity units and the departments and clinics of obstetrics and gynaecology and the four hospital trusts in northern Norway.

Norway has a population of 5 million inhabitants and is divided into four health regions (southeast, western, central and northern region). The northern region has only 9.4% of the total population and people are scattered within an area of 112,946 km^2^. To serve the population, the Northern Norway Regional Health Authority (NNRHA) trust runs six midwife managed maternity units (MAMU) (Brønnøysund, Mosjøen, Nordland hospital (NH) Gravdal, Lenvik, Sonjatun, Alta), seven departments of obstetrics and gynaecology (DoOG) [Helgeland hospital (HH) Sandnessjøen, HH Rana, Nordland hospital (NH) Vesterålen, University hospital of north Norway (UNN) Harstad, UNN Narvik, Finnmark hospital (FH) Hammerfest, FH Kirkenes] and two regional specialist hospitals (RSH) (NH Bodø, UNN Tromsø). An overview is shown in Figure 1. The decision concerning level of care is generally carried out early during pregnancy according to national guidelines. Possible occasions are the follow up visit in primary health care during the first trimester or at ultrasound screening at 18^th^ week of pregnancy.

### Data included

In Norway, all births are reported to the Medical Birth Registry of Norway (MBRN) (http://www.fhi.no/mfr). The registry was established in 1967 and was organized in the wake of the thalidomide catastrophe. The particular aim was epidemiological surveillance of birth defects and other perinatal health problems in order to detect, as soon as possible, any future increase in rates. Today it is a national health registry containing information about all births in Norway. The registry aim to clarify the causes and consequences of health problems related to pregnancy and birth, as well as to monitor the incidence of congenital abnormalities. Data for the last years are available online from the MBRN database (http://www.fhi.no/mfr). In this study, we accessed data (as of March 2012) reported from the 15 institutions in northern Norway and cumulative data from all institutions in Norway. The figures for the three year time period 2009–2011 were analyzed. The following was accessed:

•Births, newborns (only data for 2009–10), born alive or dead, weight below 2.5 kg and Apgar score <7 five minutes after birth, forceps and vacuum assisted delivery [5,6].

•The frequency of multiple pregnancies, pregnancy induced diabetes, eclampsia, perineal rupture grade 3 (partial or total tear through the anal sphincter) or 4 (grade 3 with extension through the rectal mucosa) and caesarean section.

The mentioned outcomes were chosen to describe the quality of care as they were available from the MBRN’s online databank (http://www.fhi.no/mfr). Other factors as birth weight, atonic uterus, bleeding 500–1500 ml, bleeding >1500 ml and transfusion are parameters registered by the institutions. Unfortunately, these data were, as mentioned, not available from the online databank and consequently not included in our study.

The Norwegian Surveillance Program for Infections in Hospitals (NOIS) (http://www.fhi.no/nois) was established in 2005. In this registry, surgical wound infections within 30 days after caesarean section are registered prospectively annually from Sept. 1 to Nov. 30. A total of 39 hospitals (9 in northern Norway) participated in the national survey program. Women were followed up after surgery by mail and a phone call from specialized nurses. Infection within 30 days after surgery were registered. Superficial infections could be reported by the women themselves, but all other infections had to be confirmed by a medical doctor. We collected these data for the time period 2009–2011.

### Quality control, statistical analysis and authorisation

Individual data were recorded and analyzed by the MBRN. The quality assurance of the primary data, included linking to the National Population Register (NPR), was performed to identify and confirm the women and collect available information about date of birth and death (in case of death). Furthermore, the reporting institutions were requested to supply additional information when needed. We accessed anonymous and aggregated data from this open source. Similarly data were accessed from the NOIS-registry. The aggregated data were imported to a database at the NNRHA. Microsoft Excel 2007 version was used for the final database, calculations and statistical analysis. The comparison between institutions with regard to quality of care figures was based on rates. Descriptive statistics and the t-test were used for the comparison between institutions. Significance was set to 5%. The t-test was carried out two-sided. Data from the MBRN was available on the Web free of cost and as we imported only aggregated data, no ethical committee or Data Inspectorate approval was necessary. Consequently no approval from the Regional Committees for Medical and Health Research Ethics (REK) was necessary. Similarly, no approval from the Norwegian Social Science Data Services (NSD) was required.

## Results

**Figure 2:**
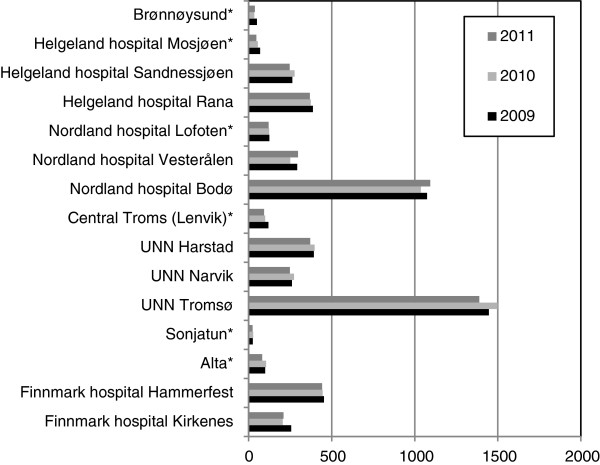
The figure shows the number of deliveries in 2009–2011 according to the 15 delivery units in northern Norway.

During the study period there were in total 15,586 births in northern Norway. This accounted for 8.6% of all births in Norway. Multiple pregnancies were significantly less common (P = 0.02) in northern Norway. The mean percentage was 1.3% (range 1.2 – 1.5%) and 1.7% (range 1.7-1.8%) in the northern region and Norway, respectively. No twins were born in midwife administered maternity units in northern Norway. The annual figures (2009–2011) at the 15 northern maternity units are shown in Figure 2. There were 1,332 births (8.5%) at MAMUs, 6,712 births (43.1%) at DoOGs and 7,539 births (48.4%) at RHSCOGs during study period.

**Figure 3:**
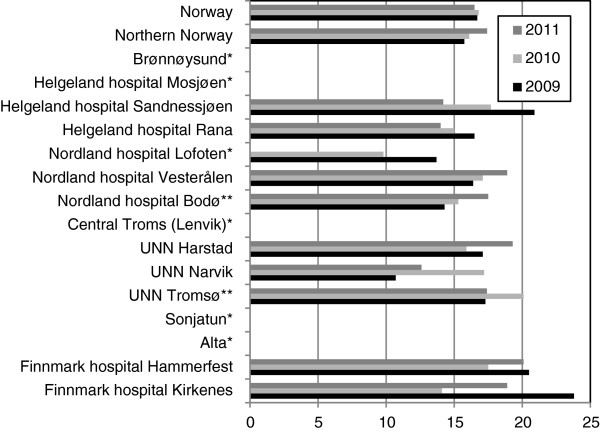
The figure shows the national data and the percentage of caesarean section (all Robson groups) at the various institutions in northern Norway.

The frequency (all Robson groups) of caesarean section was 16.4% in northern Norway (Norway 16.7%) (*P* = 0.72). The range within the region is shown in Figure 3. Except for NH-Lofoten, no MAMU had any caesarean section performed. [Due to distance to nearest DoOG and rough weather conditions, especially during winter times, NH-Lofoten has a gynaecologist on duty who may perform caesarean section]. Somewhat surprising, the highest frequency of caesarean section was not observed in the regional specialist hospitals, but in the Finnmark hospital (FH) (FH Hammerfest 19.4% and FH Kirkenes 18.9%). However, the difference did not reach statistical significance (*P* = 0.10) and the percentage in the Finnmark hospital trust (three locations: Alta, Kirkenes and Hammerfest) was only 16.9%.

The use of forceps delivery was significantly lower in northern Norway than the national figure. Whereas it was employed in 0.3% of births in our region, the Norwegian figure was almost six times higher (1.7%) (*P* < 0.01). None of the northern institutions reached the national level (highest NH Vesterålen 0.8%). Vacuum assisted delivery was also less common in our region (6.4% versus 8.3%) (*P* = 0.01). Only one of the northern institutions reached the national level. Details are shown in Table 1.

**Table 1:** Number of births, stillbirths and other birth associated factors in northern Norway and Norway in the time period 2009–2011

**Institution**	**Births**	**Caesarean (%)**	**Vacuum assisted (%)**	**Forceps delivery (%)**	**Apgar score < 7 (%)**	**Weight < 2500 g (%)**	**Still-birth per 1000**	**Perineal rupture G 3–4 (%)**	**Eclampsia per 1000**	**Pregancy induced diabetes per 1000**
FH Kirkenes	671	18,9	8,1	0,5	0,9	2,4	1,6	1,2	0,0	9,1
FH Hammerfest	1342	19,4	5,5	1,5	2,7	4,2	2,8	1,8	1,4	21,5
Alta*	286	0,0	0,0	0,3	1,1	0,7	0,0	0,7	0,0	11,6
Sonjatun*	76	0,0	0,0	0,0	1,4	1,4	0,0	1,3	0,0	26,2
UNN Tromsø	4334	18,3	5,2	1,0	2,9	7,0	3,3	1,6	0,5	15,5
UNN Narvik	782	13,5	6,0	0,4	1,1	1,5	3,6	1,1	2,4	13,5
UNN Harstad	1160	17,4	6,7	1,0	1,2	2,4	2,5	2,0	0,8	12,3
Lenvik*	312	0,0	0,0	0,3	1,0	0,7	0,0	1,6	0,0	0,0
NH Bodø	3205	15,7	8,9	1,0	1,7	7,5	4,3	1,9	0,8	28,6
NH Vesterålen	841	17,5	4,4	1,1	1,8	2,5	4,5	2,3	0,0	10,6
NH Lofoten*	367	11,8	2,7	0,5	1,4	1,9	0,0	3,4	0,0	8,2
HH Rana	1129	15,2	8,0	1,0	1,8	1,9	2,6	2,4	0,9	9,1
HH Sandnessjøen	787	17,6	7,7	0,8	2,5	2,2	6,1	1,4	2,4	14,4
HH Mosjøen*	170	0,0	0,0	0,0	0,0	0,5	0,0	0,6	0,0	14,8
Brønnøysund*	121	0,0	0,0	0,0	0,0	0,0	0,0	0,0	0,0	0,0
Northern Norway	15586	16,4	6,6	0,9	2,1	4,7	3,4	1,5	1,1	17,4
Norway	180829	16,7	8,3	1,7	1,8	5,0	3,4	2,3	0,7	17,5

*FH* = Finnmark hospital, *UNN* = University hospital of North Norway, *NH* = Nordland hospital, *HH* = Helgeland hospital. * = Midwife administered maternity unit.

Apgar scores below 7 are considered fairly low (critically low < 4). The percentage of Apgar score < 7 (measured five minutes after birth) was similar (2.1 versus 1.8%) to the national figure (*P* = 0.19). Due to selection, the three hospitals (FH Hammerfest, UNN Tromsø and NH Bodø) with a paediatric unit had consequently more newborns with lower Apgar score than the others in northern Norway (*P* < 0.01). The number of newborns with a birth weight below 2.5 kilo was significantly higher in our region than in Norway (4.7 vs. 5.0%) (*P* < 0.04). Generally, these babies should be born in regional specialist hospitals or local hospital departments of obstetrics and gynaecology. Consequently, a significant difference was confirmed comparing these units and the MAMUs (*P* < 0.02). Together with the fact that no twins were born in MAMUs and hospitals with a paediatric unit had significantly more newborns with lower Apgar score, this strongly indicated that the selection process was efficient. Details are shown in Table 1.

The stillbirth rate per 1,000 births was the same in the northern region as in Norway (3.4 stillbirths/1,000 births) (*P* = 0.80). This may indicate a similar quality of healthcare during pregnancy in northern Norway as in the other parts of the country. There were no differences between RHSCOGs and DoOGs (P = 0.75). Looking at perineal rupture grade 3 and 4, our region had a lower figure (1.5% vs. 2.3%) (*P* < 0.02) and within the northern region there was no difference between MAMUs and the others (1.3% vs. 1.7%) (*P* = 0.28). All figures are shown in Table 1.

Eclampsia was not more common in northern Norway (1.1 vs. 0.7 per 1,000) (*P* = 0.12) and within the region there was no difference between the RHSCOGs and DoOGs (*P* = 0.54). The highest figure (2.4‰) was revealed in two minor hospitals (Narvik and Sandnessjøen), but these figures must be handled with caution due to low numbers. Details are shown in Table 1.

The frequency of pregnancy induced diabetes was similar in our region as in Norway (17.4 vs. 17.5 per 1,000) (*P* = 0.99). Statistical analyses did not detect any significant difference between MAMUs and DoOGs (*P* = 0.51) or between RHSCOGs and DoOGs (*P* = 0.07).

Looking at the incidence of post-caesarean surgical wound infections, the incidence was higher in northern Norway (10.5% vs. 7.4%) (*P* < 0.01). Details are shown in Table 2. The most striking finding was the difference in rates between the two major regional specialist hospitals in the region (1.5% - NH Bodø, 17.2% UNN Tromsø).

**Table 2:** Number of infections following caesarean section during September - November in 2009–2011

**Institution**	**Number of caesarean section**	**Number of infections**	
		**No**	**(%)**
HH Rana	35	4	(11.4%)
FH Hammerfest	71	13	(18.3%)
FH Kirkenes	33	2	(6.1%)
NH Bodø	133	2	(1.5%)
NH Lofoten	7	0	(0%)
NH Vesterålen	25	0	(0%)
UNN Harstad	54	2	(3.7%)
UNN Narvik	20	3	(15.0%)
UNN Tromsø	203	35	(17.2%)
Northern Norway	581	61	(10.5%)
Norway	6637	489	(7.4%)

## Discussion

We have indicated that women in northern Norway were offered a similar quality of obstetric care as the one offered to Norwegians in general. Selection criteria with regard to place of delivery seemed to work efficiently. However, there were differences. The use of vacuum and forceps assisted delivery was less common in the northern region. Furthermore, there was revealed significantly more infants with low birth weight (< 2.5 kilo) and a higher incidence of post-caesarean surgical wound infection in the northern region.

This study has both limitations and strengths. As mentioned in the methods section, more outcomes could beneficially been included. Furthermore, we analysed aggregated data both from the MBRN and the NOIS database. Consequently, sub-analyses on specific subgroups could not be performed. We analysed a short time period (2009–2011). Trends could therefore not be looked at. The strength of this study is that it included all birth in Norway and Northern Norway. By law Norwegian medical institutions and doctors must report every birth. This fundament of the MBRN is one of the reasons for their good quality of data. The fact that the MBRN data are reported in annual reports and as easily available online services to institutions and medical doctors may improve quality and strengthen the reporting doctors’/institutions’ ownership and interest in delivering high quality data.

The caesarean section rate was 16.4%. Within Norway, the lowest figure (12%) was reported from the Western region (Hordaland county) [7]. The national figures have been rising during the last decade (2000–2010) from 13.6% to 17.1% [7]. In England, Scotland, Finland, Sweden and Denmark figures have been reported rising from around 4-5% in 1970 to 20-22% in 2001 [8]. Today, across Europe figures vary widely from about 14% in Nordic countries to 40% in Italy [9]. Avoidance of unnecessary caesarean section has been a quality target and was one among several topics at the International Forum on Quality & Safety in Healthcare’s meeting in Paris last year [3,10]. Overuse of caesarean section exposes both the mother and the fetus to unnecessary risks. To counteract the rise, policy related interventions include the requirement for a second obstetric opinion, education of health professionals, patient and community education, clinical audit and feedback mechanism, clinical practice guidelines, quality improvements strategies and financial incentives [8,10]. The benefits of patient decision aids in obstetrics have been explored [11]. When aiming for a reduction in caesarean delivery rate, the trends needs to be monitored carefully in order to prevent a shift from planned to emergency caesarean delivery, as the latter has additional delivery-related risk factors [12].

We revealed an increased risk of low birth weight infants in northern Norway. The association between maternal smoking and hypertension and low birth weight infants has been well established [13,14]. Whereas 18.5% of pregnant women in Norway were reported smokers, the figure in the northern region was 26.9% (range between counties 25.4% - 29.0%) [7]. Looking at hypertension, 52.0‰ of the pregnant women in Norway experienced pregnancy induced hypertension in 2010 [7]. The corresponding figure in northern Norway was also 52.0‰. Consequently, maternal smoking habits may be the main culprit of low birth weight in northern Norway. However, there was no notable difference in Apgar score < 7.

In this study, we disclosed a low rate (1.5%) of severe perineal rupture in the northern region. Investigators have reported incidence figures of 1-5% [15,16]. Risk factors for severe perineal tears are maternal age, parity, race, instrument assistance, episiotomy, birth weight and shoulder dystocia [15]. Looking at our data, we had less use of instrument assistance and more children with low birth weight, This could explain the low rate of severe perineal ruptures in our region.

Multiple births from assisted reproductive technology (ART) have been reported accounting for a substantial proportion of twins and triplets and higher order infants [17]. The number of multiple pregnancies in the ART-setting in Norway reduced from 1999 to 2010 from 26.5% to 10.1% [7]. Whereas 8.6% of all births in Norway occurred in northern Norway, only 3.9% of infants conceived by IVF were “made” in this region. The low number of infants conceived by IVF in the northern region may be one explanation for why fewer twins were born in this region.

With regards place of birth in Northern Norway, prematurity is in general the major factor causing altered place of delivery. The new updated selection criteria identified that increasing body mass index (BMI), pregnancy induced diabetes mellitus and previous caesarean section were other major factors in determining place of birth [3]. In common with other developed countries, these diseases are a growing problem in Norway [18]. In our analysis pregnancy related diabetes was 17.4%.

Postoperative infection following caesarean section is common and was more frequent in our region. A Norwegian study from 2009 revealed that one in 12 women (8.3%) undergoing caesarean section experienced postoperative infection [19]. The risk was significantly higher among women aged above 29 years. Most infections (86%) were disclosed after hospitalization by the primary health care or the women themselves. The figures were similar or better than reports from other European countries [19]. The significantly higher figures in northern Norway should be investigated. The continuous registration (no longer only 3 months/year) was implemented in late 2012 and more robust data will soon be available. The recent improvement project in Baerum Hospital may be of interest in this setting [20]. They managed to reduce the post-caesarean surgical wound infection rate from 17.4% to 3.1%. The following areas were focused: Preoperative hair removal, suture for skin closing, dressing in the operation room, wound dressing, double gloving, preoperative surgical hand wash and aseptic techniques.

Neonatal mortality or maternal mortality was not monitored in our survey. The Norwegian figure of neonatal mortality was not available at hospital trust level. According to the MBRN (http://www.mfr.no), the neonatal mortality rate per 1,000 births in the time period 2002–2011 was 2.3 and 2.2 in Norway and northern Norway, respectively. The corresponding late neonatal mortality rate (7–27 days after delivery) figures were 0.5 and 0.6 respectively. Maternal mortality ratio (MMR) has been analyzed in 22 European countries [21]. The overall figures were 6.3 per 100,000 live births (range 0–29.6). The Norwegian figure in this survey was 3.5. This strongly indicated that maternal death is occurring very rare and consequently not a suitable marker to analyze within the short time interval of our study.

The main characteristic of obstetric service in northern Norway was many delivery units serving a small and scattered population. Following the new national guideline stating that there should be at least four positions for obstetricians at each department, allocation of resources should be considered to cover the extra cost [3]. Especially the high number of units in the southern region (Helgeland) should be analysed. A recent Dutch study has shown that longer travel time from the home to a hospital maternity unit was associated with an increased incidence of intrapartum/neonatal mortality and adverse outcome [22]. This may indicate that our decentralized organization model may be preferable. However, it calls for an efficient selection, updated transport protocols and a safe process. An excellent air-ambulance (fixed wing) and helicopter (rotor wing) service may bring the patients to the correct place of delivery, even when there is a short time left to delivery [23]. These resources may also bring with them a midwife or an obstetrician. To meet people’s expectations, the NNRHA trust has 11 air ambulance resources [6 planes, 2 ambulance helicopters and 3 search and rescue helicopters] scattered (7 locations) within the region. We had no data clarifying the number of deliveries occurring where they were originally planned. However, bad weather conditions may obviously have changed some plans. Most of northern Norway is located above the Arctic Circle and consequently experience seasonable darkness with no sun for up to four months (Svalbard). Cold and rough weather conditions with snow may also influence on the possibility of air-ambulance and helicopter operations due to risk of icing and landing problems [23]. In India, such a study documented that one third of women delivered at other than their planned place of delivery [24].

In this analysis, we concluded that a national registry (MBRN) was a good surveillance tool to monitor quality of care. A similar conclusion has been given by several investigators [25,26]. Shapiro-Mendoza and colleagues in Atlanta GA, USA reported the sudden unexpected infant death case registry a method to improve surveillance [25]. The surveillance system may improve researchers and program planners’ ability to create prevention strategies and interventions. Consequently, sudden unexpected infant deaths and injury-related infant deaths may be avoided [25]. The Danish National Indicator Project developed and implemented recently a set of national indicators in their registry [26].

Instruments (screening tools and checklists) are mandatory for the surveillance of quality of care. In the struggle for improved patient safety in midwifery care, Martijn and colleagues identified five domains of patient risk [27]. They were organization, communication, patient-related risk factors, clinical management and outcomes. Based on these, they developed a 32 item screening instrument. They concluded the instrument valid and feasible to assess patient safety and may be used for quantitative analysis of patient records and to identify unsafe situations. The Hospital Corporation of America recently presented their obstetric patient safety efforts and results [28]. Standardization and documentation of critical processes, establishment of national quality benchmarks, reduction in elective deliveries before 39 weeks gestation and reduction in fatal post-caesarean pulmonary embolism were the major areas of progress. A safety culture in the maternity units is important. Raftopoulos and colleagues investigated this in Cyprus [29]. A safety attitude questionnaire was employed and 140 midwives were included in the study. They revealed the highest mean scores on team work and safety climate among the more experienced group of midwives. This could indicate that younger midwives should be focused when efforts are made to improve safety culture.

Today, several European countries run patient safety programs. In Norway, a patient safety campaign called “in safe hands” has been launched. Safe surgery and fewer infections are among the goals. In the UK, Institute for Healthcare Improvement (IHI) has been an important organisation in this setting. Their annual conference has been an important tool in obtaining a focus on quality in maternity care among health care workers and administrators. In example, the IHI Scottish Patient Safety Programme (SPSP) recently implemented their Maternity Care Quality Improvement Collaborative (McQIC). (http://www.scottishpatientsafetyprogramme.scot.nhs.uk/programme/events/McQIC). Hopefully, patient safety culture and focus will reach all European delivery units and further improve the quality of care.

## Conclusion

The Northern Norway Regional Health Authority provides good quality obstetric health care. The differences in birth weight, multiple pregnancies, post-caesarean section wound infections and severe perinatal rupture between Northern Norway and Norwegian should be further elucidated and monitored in the future. Common selection criteria and a national registry are important issues when quality of obstetric care and safety are focused.

## Abbreviations

RSH: Regional specialist hospital; DoOG: Department of obstetrics and gynaecology; IHI: Institute of healthcare improvement; MAMU: Midwife administered maternity unit; MBRN: Medical birth registry of Norway; NNRHA: Northern Norway Regional Health Authority.

## Competing interests

The authors declare that they have no competing interests.

## Authors’ contributions

JN, AH and TS initiated the study and designed the protocol. BH, JN, AH, TS, FM and RE participated in acquisition of data. The final database was made by JN and TS. Statistical analysis was done by JN. Suggested sub-analyses, interpretation of data and comments with regard to the results were given by all authors. All study participants have participated in the writing process of the manuscripts and given their approval of the final version.

## Pre-publication history

The pre-publication history for this paper can be accessed here:

http://www.biomedcentral.com/1471-2393/13/175/prepub
